# Neurophysiological markers in community-dwelling older adults with mild cognitive impairment: an EEG study

**DOI:** 10.1186/s13195-023-01368-6

**Published:** 2023-12-15

**Authors:** Osamu Katayama, Yaakov Stern, Christian Habeck, Sangyoon Lee, Kenji Harada, Keitaro Makino, Kouki Tomida, Masanori Morikawa, Ryo Yamaguchi, Chiharu Nishijima, Yuka Misu, Kazuya Fujii, Takayuki Kodama, Hiroyuki Shimada

**Affiliations:** 1 Department of Preventive Gerontology, Center for Gerontology and Social Science, National Center for Geriatrics and Gerontology, 7-430 Morioka-Cho, Obu City, Aichi 474-8511 Japan; 2 Japan Society for the Promotion of Science, Chiyoda-Ku, Tokyo, 102-0083 Japan; 3 Columbia University Vagelos College of Physicians and Surgeons, New York, NY 10032 USA; 4 Department of Physical Therapy, Graduate School of Health Sciences, Kyoto Tachibana University, 34 Yamada-Cho, Oyake, Yamashina-Ku, Kyoto 607-8175 Japan

**Keywords:** Mild cognitive impairment, Community-dwelling older adults, Electroencephalography, eLORETA-ICA, Accuracy

## Abstract

**Background:**

Neurodegeneration and structural changes in the brain due to amyloid deposition have been observed even in individuals with mild cognitive impairment (MCI). EEG measurement is considered an effective tool because it is noninvasive, has few restrictions on the measurement environment, and is simple and easy to use. In this study, we investigated the neurophysiological characteristics of community-dwelling older adults with MCI using EEG.

**Methods:**

Demographic characteristics, cognitive function, physical function, resting-state MRI and electroencephalogram (rs-EEG), event-related potentials (ERPs) during Simon tasks, and task proportion of correct responses and reaction times (RTs) were obtained from 402 healthy controls (HC) and 47 MCI participants. We introduced exact low-resolution brain electromagnetic tomography-independent component analysis (eLORETA-ICA) to assess the rs-EEG network in community-dwelling older adults with MCI.

**Results:**

A lower proportion of correct responses to the Simon task and slower RTs were observed in the MCI group (*p* < 0.01). Despite no difference in brain volume between the HC and MCI groups, significant decreases in dorsal attention network (DAN) activity (*p* < 0.05) and N2 amplitude of ERP (*p* < 0.001) were observed in the MCI group. Moreover, DAN activity demonstrated a correlation with education (Rs = 0.32, *p* = 0.027), global cognitive function (Rs = 0.32, *p* = 0.030), and processing speed (Rs = 0.37, *p* = 0.010) in the MCI group. The discrimination accuracy for MCI with the addition of the eLORETA-ICA network ranged from 0.7817 to 0.7929, and the area under the curve ranged from 0.8492 to 0.8495.

**Conclusions:**

The eLORETA-ICA approach of rs-EEG using noninvasive and relatively inexpensive EEG demonstrates specific changes in elders with MCI. It may provide a simple and valid assessment method with few restrictions on the measurement environment and may be useful for early detection of MCI in community-dwelling older adults.

**Supplementary Information:**

The online version contains supplementary material available at 10.1186/s13195-023-01368-6.

## Background

Mild cognitive impairment (MCI) is characterized by cognitive dysfunction that does not significantly interfere with independent living and may be a transitional stage between normal cognitive aging and Alzheimer’s disease (AD) [1, 2]. It has been reported that as AD progresses in individuals with MCI, a gradual decline in cognitive function spreads to other domains [3]. A systematic meta-analysis of the literature reporting brain structural changes associated with MCI found a 2.2-fold decrease in volume in the hippocampus, 1.8-fold in the whole brain, and 1.5-fold in the entorhinal cortex in individuals with MCI [4]. AD is a neurodegenerative disorder characterized by the deposition of amyloid and tau proteins [5]. The accumulation of amyloid and tau may precede the onset of cognitive symptoms by several years and is thought to gradually lead to changes in brain structure and the onset of clinical manifestations [5, 6]. It has been shown that amyloid beta (Aβ) abnormalities temporally precede brain structural changes in older adults with MCI [7, 8]. The incidence of dementia in persons with MCI is estimated to be 5–15% per year [5, 9]. It is important to characterize the state of neural activity in the brain during the period before structural brain changes are observed in patients with MCI. Previous studies using resting-state functional magnetic resonance imaging (rs-fMRI) to evaluate regional brain interactions have found differences in functional connectivity between healthy controls (HC) and patients with MCI [10, 11].

Research on MCI is also being conducted using electroencephalography (EEG), which is non-invasive and relatively inexpensive and allows for measurements with minimal spatial constraints. Studies using EEG often focus on measuring event-related potentials (ERPs), taking advantage of the temporal resolution, which is a prominent feature of brain waves [12]. ERPs consist of different components, including positive and negative components, which occur over time after stimulus onset, and each has a different meaning [13, 14]. Among these, the N2 component, which has a negative peak around 200 ms after stimulus presentation, is thought to reflect discrepancies in cognitive control processes and information processing [15, 16], whereas the P3 component, which has a positive peak after 300 ms, is associated with error detection and information processing for forecasts [17, 18]. In the Simon task, participants respond to the symbolic features of the stimulus (e.g., shape or color) using either their left or right hand. Because the stimulus is presented on the left or right side, the stimulus location may or may not be spatially congruent with the target’s responding hand [19]. Thus, the Simon task can measure visuospatial processing and execution (response-related) processes of lateralized stimuli. Performance is typically facilitated by congruent stimulus locations and inhibited by incongruent stimulus locations [20]. Therefore, the N2 and P3 components have been extensively investigated in previous studies on MCI using the Simon task [21, 22], which is a choice reaction task [20, 23]. Source estimation software has been developed to overcome the spatial resolution limitations of EEG [24, 25]. With the advancement of this technology, EEG can now capture connectivity between distant regions similar to fMRI [26, 27]. In resting-state EEG (rs-EEG) connectivity analysis, in addition to frequency-specific analysis, cross-frequency network analysis, such as exact low-resolution brain electromagnetic tomography-independent component analysis (eLORETA-ICA), has become possible, and reports have begun to emerge on healthy adults [28], patients with dementia with Lewy bodies [29], and individuals with MCI have begun to emerge [30, 31].

The growing awareness of brain health and AD in the general population is leading to an increase in the number of cognitively impaired individuals who are concerned that they have reduced cognitive function and who are seeking help from the medical system [32]. However, medical attention may be delayed in older adults because they do not perceive significant changes in their daily functioning or quality of life [33], consider their impairment to be due to normal aging rather than pathological brain dysfunction [34], and are less likely to compare their cognitive abilities with those of others. Therefore, older adults with MCI in the community may include those with delayed detection of cognitive decline and progression. Further, it is not easy for community-dwelling individuals with MCI to undergo 18F-florbetapir PET imaging, measurement of Aβ in cerebrospinal fluid, and MRI at medical facilities. Therefore, EEG may contribute to the early detection of MCI in community-dwelling individuals with possible MCI. This may validate the underlying AD-related brain changes in patients with MCI instead of using more expensive PET scans.

In this study, we performed EEG measurements at rest and during cognitive tasks in community-dwelling older adults, both healthy and with MCI to capture the neurophysiological changes associated with MCI. We used the recently developed eLORETA-ICA analysis, which allows network analysis across frequencies, in addition to ERP analysis and brain volume measurements using MRI. In addition, we comprehensively collected data by conducting interviews regarding medical history, physical function examinations, cognitive function assessments, and measurement of behavioral indicators during cognitive tasks. It has been suggested that differences in the efficiency and capacity of brain networks may explain individual differences in cognitive performance as well as individual differences in the ability to cope with brain changes. This concept of individual brain characteristics is referred to as the cognitive reserve (CR) [35]. Studies comparing HC and MCI have shown differences in brain volume in longitudinal MRI studies [4], differences in eLORETA-ICA in rs-EEG [30], and differences in ERP [21]. We hypothesized that when comparing adults with MCI and HC living independently in the community, they would not show differences in brain volume, but those with MCI would show cognitive impairments and differences in the network of rs-EEG and ERP during cognitive tasks. Confirming this hypothesis would further support the efficacy of EEG for the early detection of MCI and functional assessment in community-dwelling older adults at a potential risk of developing AD.

## Methods

### Participants

A total of 449 participants (249 female, median age 73, interquartile range [IQR] 69–78) were recruited from an ongoing study of the National Center for Geriatrics and Gerontology-Study of Geriatric Syndromes (NCGG-SGS) [36] on health promotion for older adults in the Aichi prefecture in Japan. The NCGG-SGS is a cohort study aimed at establishing a screening system for geriatric syndromes and validating evidence-based interventions to prevent them. The study protocol was approved by the ethics committee of the National Center for Geriatrics and Gerontology (Approval Number: 1440–5). This study was conducted in accordance with the principles of the Declaration of Helsinki. All participants provided written informed consent prior to inclusion. Participants were recruited from the “Self-Management Activity Program for the Older” study, which examines the effects of behavioral modification techniques on the prevention of dementia among community-dwelling older adults using a smartphone as a behavior change tool [37].

The health check procedure included a detailed evaluation by a nurse to exclude individuals with significant or unstable medical conditions, as well as those with a significant neurological history (such as epilepsy, brain tumors, or stroke). We used the Edinburgh Handedness Inventory (EHI) score to categorize right- and left-handed dominance. In addition, participants’ cognitive function was evaluated using the Mini-Mental State Examination (MMSE) [38] and the NCGG-Functional Assessment Tool (NCGG-FAT) [39–41], which comprises four domains: (1) memory (word list memory I [immediate recognition] and word list memory II [delayed recall]); (2) attention (a tablet version of Trail Making Test Part A: TMT-A); (3) executive function (a tablet version of Trail Making Test Part B: TMT-B); and (4) processing speed (a tablet version of the symbol digit substitution test: SDST). Participants included 402 cognitively intact participants (229 female; median age, 73 years; IQR 70–78 years) and 47 participants with documented MCI (20 female; median age, 73 years; IQR, 69–78 years). None of the participants received medications for MCI at a medical facility. The nurse confirmed during the face-to-face interview that the participant was not taking any medication for MCI. However, the MCI participants were taking medications for other conditions (heart disease, diabetes, hypertension, and hyperlipidemia). We found no significant difference in the number of medications taken between HC and MCI participants. We did not restrict medications for other conditions prior to EEG measurement for risk management purposes.

Demographic characteristics are presented in Table 1.

**Table 1:** Demographic characteristics

	**Total**	**HC**	**MCI**	***p* value**
	*n* = 449	*n* = 402	*n* = 47	
**Variable**				
Age, year (IQR)	73 (69–78)	73 (70–78)	73 (69–78)	0.915
Sex, woman (%)	249 (55.5)	229 (57.0)	20 (42.6)	0.084
EHI total, score (IQR)	100 (100–100)	100 (100–100)	100 (100–100)	0.843
Handedness, right (%)	436 (97.1)	390 (97.0)	46 (97.9)	1.000
Education, year (IQR)	12 (12–16)	12 (12–16)	12 (12–14)	0.492
Heart disease, yes (%)	57 (12.7)	48 (11.9)	9 (19.1)	0.241
Diabetes, yes (%)	64 (14.3)	61 (15.2)	3 (6.4)	0.158
Hypertension, yes (%)	170 (37.9)	156 (38.8)	14 (29.8)	0.295
Hyperlipidemia, yes (%)	157 (35.0)	146 (36.3)	11 (23.4)	0.111
Medication, n (IQR)	2 (1–4)	2 (1–4)	2 (1–4)	0.851
Grip strength, kg (IQR)	26.1 (21.7–32.9)	26.1 (21.7–32.9)	25.6 (21.4–31.9)	0.786
Walking speed, m/sec (IQR)	1.24 (1.12–1.36)	1.25 (1.12–1.37)	1.19 (1.07–1.29)	0.079
GDS, score (IQR)	2 (1–4)	2 (1–3)	2 (1–4)	0.642
Living alone, yes (%)	76 (16.9)	64 (15.9)	12 (25.5)	0.145
Work, yes (%)	101 (22.5)	88 (21.9)	13 (27.7)	0.477
**Cognitive function**				
MMSE, score (IQR)	29 (27–30)	29 (27–30)	28 (26–29)	0.002^a^
Word list memory, composite score (IQR)	12.3 (9.67–14.3)	12. 7 (10.3–14.3)	7. 7 (6.5–11.7)	< 0.001^a^
TMT–A, seconds (IQR)	18 (16–21)	18 (16–20)	22 (19.5–28.5)	< 0.001^a^
TMT–B, seconds (IQR)	31 (25–39)	31 (25–38)	39 (31.5–57)	< 0.001^a^
SDST, score (IQR)	49 (44–55)	50 (45–55)	43 (35.5–48)	< 0.001^a^
**Brain volume**				
Right Hippocampus, mm^3^ (IQR)	3916.3 (3619.4–4189.9)	3915.3 (3622.7–4194.5)	3917.5 (3596.4–4179.8)	0.896
Left Hippocampus, mm^3^ (IQR)	3745.5 (3477.2–3998.8)	3743.4 (3470. 7–3993.3)	3780.8 (3545.5–4097.4)	0.575
Cerebral White Matter, mm^3^ (IQR)	410,081.0 (380,472.0–439,302.0)	410,414.0 (379,324.8–439,100.8)	407,836.0 (387,099.0–440,727.5)	0.656
Sub Cortical Gray Matter, mm^3^ (IQR)	51,097.0 (48,069.0–54,025.0)	51,040.5 (48,046.8–53,942.8)	51,599.0 (48,304.5–54,399.0)	0.312
Total Gray Matter, mm^3^ (IQR)	562,343.6 (533,467.7–596,639.3)	560,918.7 (532,063.8–595,307.1)	575,054.9 (551,714.0–599,438.8)	0.107
eTIV, mm^3^ (IQR)	1,357,196.5 (1,264,368.5–1,463,975.8)	1,356,346.3 (1,264,522.8–1,459,575.0)	1,357,982.6 (1,273,443.8–1,530,433.9)	0.326

*HC* healthy condition, *MCI* mild cognitive impairment, *IQR* interquartile range, *EHI* Edinburgh Handedness Inventory, *GDS* 15-item Geriatric Depression Scale, *MMSE* Mini-Mental State Examination, *TMT* Trail Making Test, *SDST* Symbol Digit Substitution Test, *eTIV* estimated total intracranial volume

^a^Wilcoxon rank sum test

### Defining MCI

We followed a methodology similar to that of a previous study in which clinical, neuropsychological, and laboratory data were reviewed by neurologists to identify participants with MCI [1]. MCI was defined as cognitive impairment with a cognitive test score of 1.5 standard deviations or more below the mean in any cognitive domain, while functionally independent in activities of daily living (ADL). We used the MMSE to measure global cognitive function, with a score of < 24 indicating impairment [42]. The NCGG-FAT was used to assess specific cognitive functions including memory, attention, executive function, and processing speed. All tests have established standardized thresholds for defining the objective cognitive impairments in the corresponding domains (a score of ≥ 1.5 standard deviations below those age- and education-specific means, based on our own algorithm sourced from a database, including over 10,000 community-dwelling older adults), which were derived from a population-based cohort [43]. Participants whose cognitive test scores were > 1.5 standard deviations below the mean in all domains were classified as HC [41, 44, 45].

### Simon task procedure

**Fig. 1:**
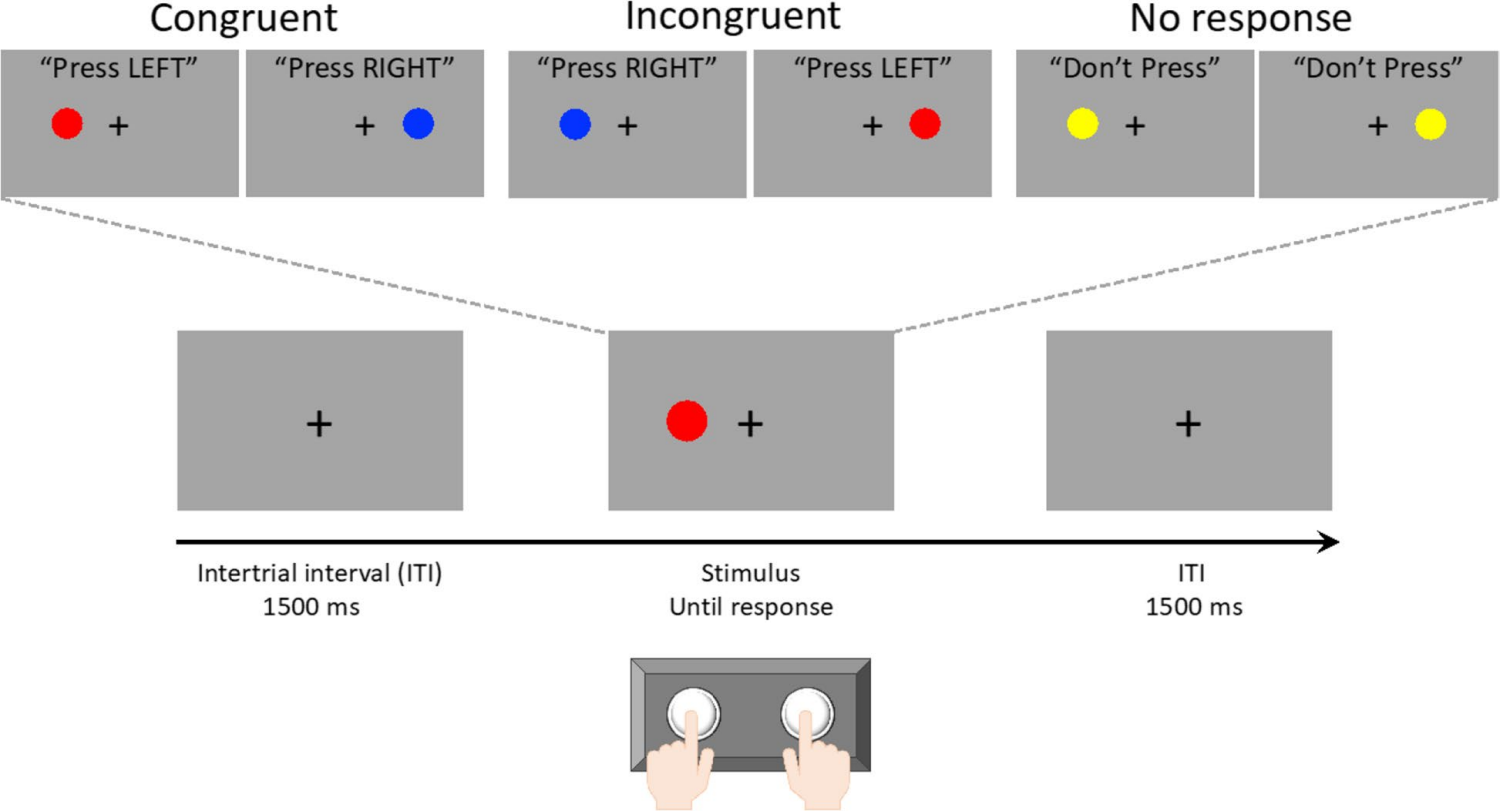
Overview of the Simon task

We investigated error processing during the Simon task, which is a choice reaction task that generates errors using incongruent spatial and color cues [20, 23]. Building on previous electrophysiological research [46], we used a modified version of the Simon task that produces a high frequency of errors. In this task, participants were presented with a colored cue to the left or right of a fixation cross (Fig. 1). The cue color determines the response direction, with blue indicating a right-handed response, red indicating a left-handed response, and yellow indicating no response. The spatial location and cue direction were either congruent or incongruent, with a prepotent response requiring inhibition in the incongruent condition (i.e., responding in the direction of the spatial location of the cue rather than the direction indicated by the color). Errors occurred when the participant’s response direction was inconsistent with the cue color. To increase the number of errors, the participants were encouraged to complete the task quickly. A total of 150 trials were randomly presented to each participant, with each condition (i.e., congruent, incongruent, and no response) consisting of 50 trials. All participants performed 24 trials (eight in each of the three conditions) for training before scanning. The proportions of correct responses and reaction times (RTs) were calculated separately for the congruent, incongruent, and no-response conditions. The intertrial interval was 1500 ms [47].

Participants responded by pressing the left or right button, depending on the color of the circle, while ignoring the position of the stimulus (left/right). Three conditions could occur: congruent, when the color and position of the circle led to the same response; incongruent, when the color and position did not lead to the same response; and no response, when the yellow circle was the color and no button should be pressed regardless of the position.

### rs-MRI acquisition and image processing and analysis

The rs-MRI was performed using a 3-T Siemens MAGNETOM Trio Tim 3 T scanner (Siemens Medical Solutions, Erlangen, Germany) with 12 channel head coils. A whole brain three-dimensional T1-weighted magnetization prepared rapid acquisition gradient echo sequence was acquired in the sagittal plane: repetition time = 1800 ms, echo time = 1.99 ms, flip angle = 9°, slices = 160, slice thickness = 1.1 mm, voxel = 1.0 × 1.0 × 1.1 mm, image matrix = 256 × 256 mm, field of view = 250 mm. Each scan took 4:06 min.

We used FreeSurfer version 7 (http://surfer.nmr.mgh.harvard.edu) on a Linux server (Ubuntu version 20.04) for image processing [48]. The automated processing stream includes the removal of non-brain tissue [49], Talairach transformation, gray/white matter tissue segmentation [50], intensity normalization, topological correction of the cortical surface [51], and surface deformation to optimize the placement of tissue borders [52]. We calculated brain volumetric measurements (mm^3^) for the right and left hippocampi, cerebral white matter (CWM), subcortical gray matter (SGM), and total gray matter (TGM).

### rs-EEG recording and analysis

The participants underwent EEG recordings in a resting state with their eyes closed for 5 min. The participants were instructed to keep their eyes closed but stay awake during the recordings. Spontaneous cortical electrical activity was recorded using a high-standard mobile dry-based 19-channel EEG-system (CGX Quick-20r; Cognionics Inc.) and sampled at 500 Hz. EEG was recorded with the electrodes positioned according to the International 10–20 system (i.e., Fp1, Fp2, F3, F4, C3, C4, P3, P4, O1, O2, F7, F8, T3, T4, T5, T6, Fz, Cz, and Pz) using an ear reference. Electrode impedances were kept below 10 kΩ. Bandpass filtering from 0.53 to 120 Hz and a 60-Hz notch filter were performed using Brain Vision Analyzer software 2.2 (Brain Products, Munich, Germany).

We investigated the EEG data using eLORETA, which is an open-source academic software available at http://www.uzh.ch/keyinst/loreta.htm [53]. The eLORETA method can estimate cortical electrical distributions from scalp electrical potentials measured at each electrode site and precisely localize any point source in the brain using unique weights in a weighted minimum-norm inverse solution. Although arbitrary distributions can be correctly localized with low spatial resolution according to the principles of linearity and superposition, the current version of eLORETA includes 6239 cortical gray matter voxels at a 5-mm spatial resolution in a realistic head model [54], and the lead field was computed using anatomic labels corresponding to Brodmann areas. Specific activity is observed in rs-EEG activity in MCI [55–57]. Therefore, we used the same frequency bands of interest (delta [2–4 Hz], theta [4–8 Hz], alpha [8–13 Hz], beta [13–30 Hz], and gamma [30–60 Hz]) which were set to the same frequency bands as in previous studies comparing healthy older adults, MCI, and AD using eLORETA-ICA [31]. Neural activity was calculated using global field power values [25].

To identify maximally spatially independent spectral components, we performed eLORETA-ICA on the eLORETA localization images using the method described by Aoki et al. [28, 29, 31], which is available in the eLORETA software. The eLORETA-ICA method can decompose non-Gaussian cortical electrical activity into independent components (ICs) in different frequency bands and is superior to other decomposition methods, such as principal component analysis or correlation analysis, using EEG data [58–60]. Moreover, eLORETA-ICA can use all frequency information from EEG data [28–31]. The technical details of eLORETA-ICA can be found in Pascual-Marqui et al. [53]. The mean localization image was initially calculated for each frequency band of each participant using the data, which were then concatenated.

ICA is a mathematical method that decomposes a mixture of signals, such as EEG and fMRI data, into ICs consisting of physiological and artifact signals. ICA offers precise decomposition of non-Gaussian data, such as cortical electrical activity, compared with other analysis methods [59]. To identify a set of maximally independent components in eLORETA spectrocortical electrical activity across a population, group ICA was applied using the eLORETA-ICA software [61]. The data matrix consisted of participants × (concatenated frequency bands and spaces [cortical voxels]). Specifically, the eLORETA-derived 5-frequency (delta, theta, alpha, beta, and gamma) source images from each participant were expressed in a voxel-by-frequency matrix format or Nv × Nf, where Nv = the total number of voxels given by eLORETA = 6239 and Nf = 5. ICA was applied to this data matrix to identify the maximally independent spectrocortical components [62, 63]. The ICs were then ordered based on total power and color-coded for each frequency band. In the color-coded map, red and blue represent the increases and decreases in power, respectively, with an increase in IC activity. It is important to note that ICA consists of two parts: the spectrocortical networks that are common to all participants and the set of “loadings” (i.e., network activities) that are specific to each participant. For a given participant, the loadings (i.e., network activities) quantified the contribution of each network to its actual spectrocortical activity. Furthermore, once the spectrocortical networks common across a large sample are available, they can be applied to any new participant’s activity, thus producing loadings (i.e., network activities) for the new participant [29]. To measure whether the activity level in any of our identified ICs significantly differed between the HC and MCI groups, the primary outcome variables were the loading values for each IC output using the ICA algorithm. In this way, the 15 resting-state networks were used to determine the “loadings” (i.e., network activities) for the HC and MCI groups.

### ERP recording and analysis

ERPs were recorded using a high-standard mobile dry-based 19-channel EEG-system (CGX Quick-20r, Cognionics Inc.) and sampled at 500 Hz. EEG was recorded with the electrodes positioned according to the International 10–20 system (i.e., Fp1, Fp2, F3, F4, C3, C4, P3, P4, O1, O2, F7, F8, T3, T4, T5, T6, Fz, Cz, and Pz) using an ear reference. Electrode impedances were kept below 10 kΩ. Subsequent processing was performed using the Brain Vision Analyzer software 2.2 (Brain Products, Munich, Germany). First, a bandpass filter ranging from 0.1 to 30 Hz and a 60-Hz notch filter were applied to the data. Raw data was inspected to eliminate technical artifacts, and periodically occurring artifacts, such as pulse artifacts and horizontal and vertical eye movements, were subsequently detected and corrected by ICA using the infomax algorithm. Following these corrections, cue-locked segments were formed for each condition. These segments began 200 ms prior to the locking point (cue onset was set to time point 0) and ended 700 ms thereafter, resulting in an overall segment length of 900 ms. An automated artifact rejection procedure was then implemented using the rejection criteria of a maximal value difference above 200 μV in a 200-ms interval or an activity below 0.5 μV in a 100-ms period. Next, a baseline correction was set to a time interval ranging from − 200 to 0 ms before averaging the segments for each condition. The same rejection criteria were used for all participants to perform the automatic artifact removal procedure. Next, a baseline correction was set to a time interval ranging from − 200 to 0 ms before averaging the segments for each condition. The three conditions were congruent, incongruent, and no response. For N2 and P3, the local maxima were quantified semi-automatically in time windows of 151–230 ms and 300–600 ms, respectively [22, 64], at electrodes Fz, Cz, and Pz, as indicated by the grand average data. The peak latency (ms) of N2 and P3 and the amplitude (μV) at that time were then calculated for each channel. The mean number of epochs removed under each condition was congruent = 18.7, standard deviation (SD) = 1.9; incongruent = 17.2, SD = 1.7; and no response = 17.0, SD = 1.7.

### Statistics

The Shapiro–Wilk test revealed that many of the variables were not normally distributed. Therefore, Bonferroni correction for the Wilcoxon rank-sum test (Wilcoxon test) was used to detect the characteristics that differed between the HC and MCI groups. Categorical variables were compared using Pearson’s chi-squared test. We used the EHI score to categorize right- and left-handed dominance and compared the RTs for the Simon task using the Wilcoxon test. Spearman’s rank correlation coefficients were used to determine the correlations and significant differences between the demographic characteristics, cognitive tests, behavioral data, and eLORETA-ICA network activities for all participants, the HC group, and the MCI group. After observing significant differences in the values of the eLORETA-ICA network activities between the HC and MCI groups, model creation and validation were conducted to discriminate MCI. First, random oversampling was used to correct for imbalances between the HC and MCI groups. Next, each eLORETA-ICA network activity that was significantly different between the HC and MCI groups was converted to a *z*-score, which was weighted using cluster analysis as a multivariate analysis. The composite score of eLORETA-ICA network activity was then calculated from the weighted *z*-score. Finally, various performance measurements are used to verify the performance of the models. We measured the performance of each model based on the accuracy, sensitivity, specificity, precision, F1 Score, and area under the receiver operating characteristic curve (AUC). The imbalance between the HC and MCI groups was addressed using the R package “Random Over-Sampling Examples (ROSE)” (version 0.0.4) [65], a bootstrap-based method that combines over- and undersampling to address sample size imbalances [66]. The combination of over- and undersampling using ROSE has been shown to significantly improve model accuracy [65]. Support vector machine (SVM) and logistic regression analyses were performed using the “caret” package (version 6.0.94) executable in R version 4.2.2 (R Foundation for Statistical Computing, Vienna, Austria), and Monte Carlo fivefold cross-validation was applied to obtain the model that identified MCI with the highest accuracy. The number of Monte Carlo fivefold cross-validation iterations was set to 100. Model 1 was a cognitive function test; Model 2 was the eLORETA-ICA network activities composite score; Model 3 was a combination of Models 1 and 2; Model 4 was a combination of Model 3 and confounding factors; Model 5 was a combination of Model 1, confounding factors, and MRI data; and Model 6 was a combination of all the factors. The significance level was set at *p* < 0.05. All analyses were performed using R version 4.2.2 (R Foundation for Statistical Computing, Vienna, Austria).

## Results

There were no significant differences in demographic characteristics or brain volumes between the groups; only cognitive function was significantly lower in the MCI group (*p* < 0.005 after Bonferroni correction) (Table 1).

### Behavior on the Simon task

**Fig. 2:**
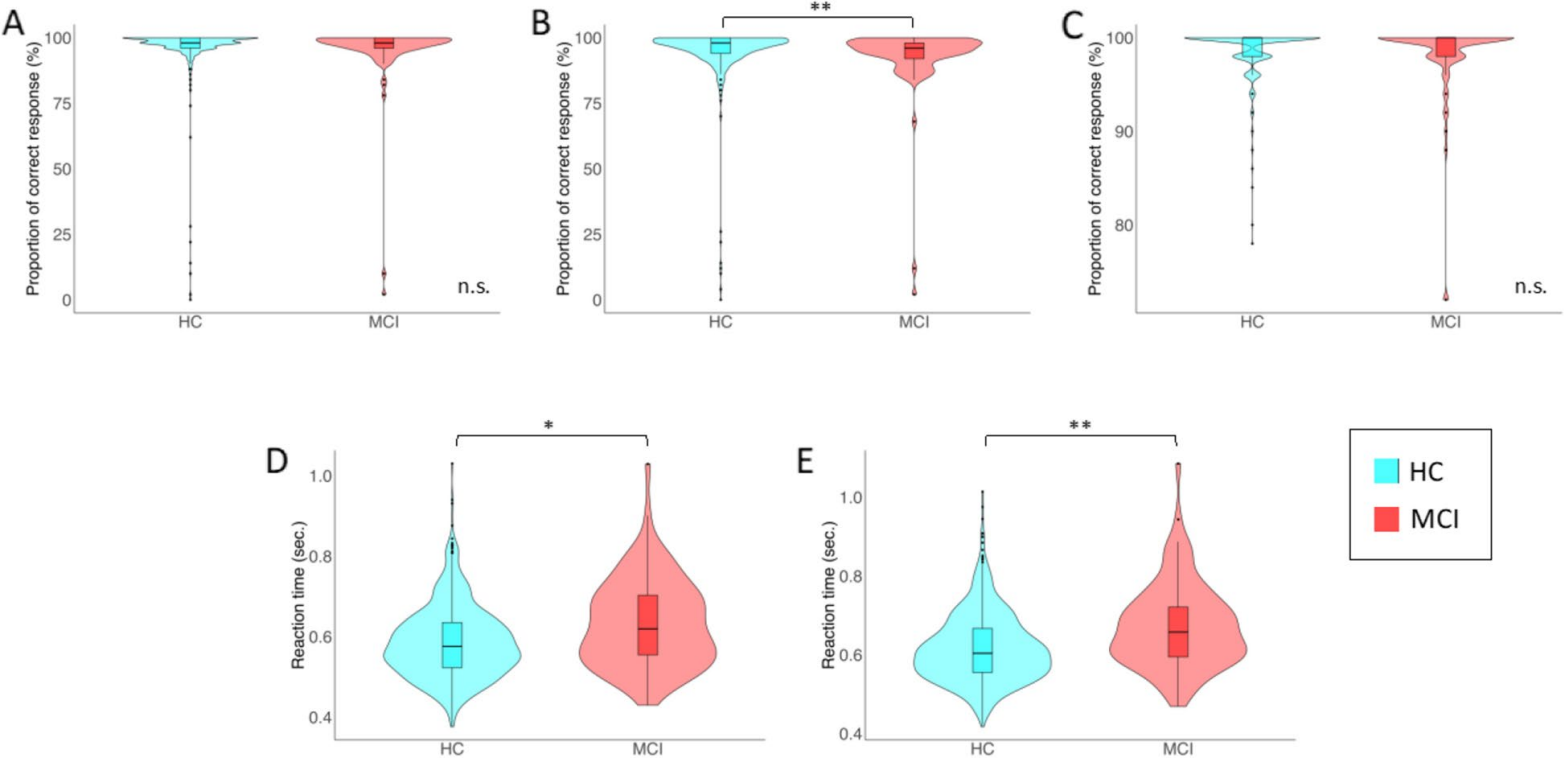
Descriptive statistics for behavioral data. **A** The proportion of correct responses in the Congruent condition. **B** The proportion of correct responses in the incongruent condition. **C** shows the proportion of correct responses in the no response condition. **D** The RT in the Congruent condition. **E** The RT in the incongruent condition. Each violin plot contains a boxplot. The black line within the box represents the median; the box in the center represents the interquartile range; the black dot depicts the remaining distribution, except for any data points identified as “outliers” (i.e., those more than 1.5 standard deviations above or below the median). HC, healthy controls; MCI, mild cognitive impairment. *p < 0.01; **p < 0.001; n.s. not significant

All participants were included in behavioral analyses (*n* = 449). The descriptive results of the behavioral data are shown in Fig. 2.

The median (IQR) proportions of correct responses in the congruent, incongruent, and no response conditions were 98% (96–100), 98% (94–100), and 100% (98–100), respectively, in the HC group, and 98% (96–100), 96% (93–98), and 100% (98–100), respectively, in the MCI group. Wilcoxon tests showed a significantly lower proportion of correct responses to the incongruent condition in the MCI group than in the HC group (*p* < 0.001). We compared the RTs for right- and left-handedness according to EHI scores and found no significant differences (*p* > 0.05). The median (IQR) RTs in the congruent and incongruent conditions were 0.574 ms (0.521–0.624) and 0.615 ms (0.537–0.703) in the HC group and 0.602 ms (0.549–0.656) and 0.653 ms (0.587–0.715) in the MCI group, respectively. Wilcoxon tests showed significantly slower RTs in the MCI group than in the HC group in both conditions (*p* < 0.01).

### eLORETA-ICA results

**Fig. 3:**
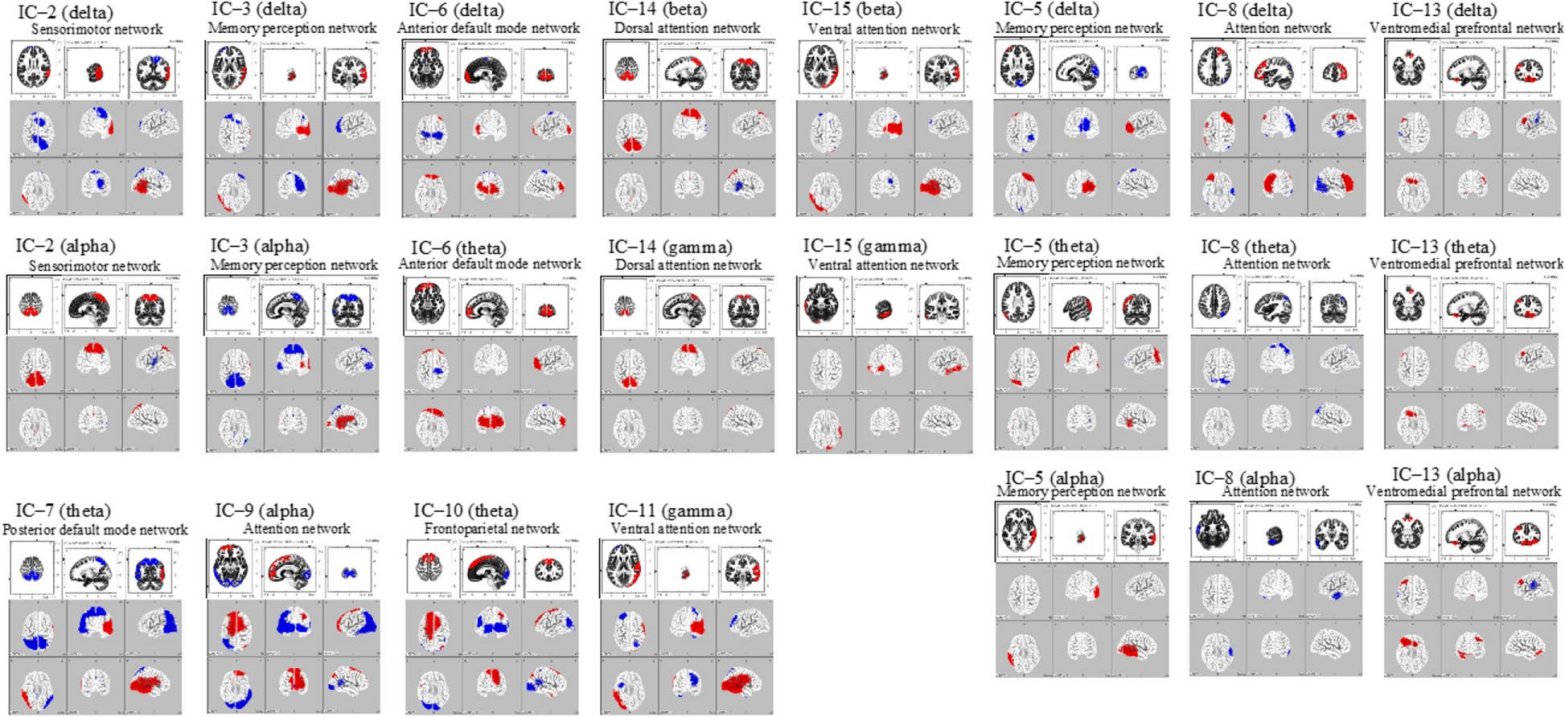
Sample images of 15 independent components (ICs) in their specified frequency bands obtained by applying eLORETA-ICA to EEG data. Twelve ICs were identified as physiological network activities: IC-2, IC-3, IC-5, IC-6, IC-7, IC-8, IC-9, IC-10, IC-11, IC-13, IC-14, and IC-15 and 3 ICs as artifact activities: IC-1, IC-4, and IC-12. In the color-coded map, red and blue represent increase and decrease in power with increasing IC activity, respectively

We applied eLORETA-ICA to rs-EEG data from 449 participants and identified 15 independent components (ICs). Twelve of them (IC-2, IC-3, IC-5, IC-6, IC-7, IC-8, IC-9, IC-10, IC-11, IC-13, IC-14, and IC-15) corresponded to physiological network activities, whereas the other three represented artifact activities (IC-1, IC-4, and IC-12) (Fig. 3). The results of eLORETA-ICA showed that out of the 12 rs-EEG networks, the MCI group showed decreased activity in the sensorimotor (SMN) (IC-2), memory perception (IC-5), posterior default mode (DMN) (IC-7), ventral attention (VAN) (IC-11), and dorsal attention (DAN) (IC-14) networks (Fig. 3). The mean ± standard deviation of the SMN, memory perception network, posterior DMN, VAN, and DAN, respectively, was 2760.3 ± 808.3 μV^2^/M^4^/Hz, 1958.0 ± 654.2 μV^2^/M^4^/Hz, 706.8 ± 994.7 μV^2^/M^4^/Hz, 696.1 ± 1191.8 μV^2^/M^4^/Hz, and 2737.9 ± 752.2 μV^2^/M^4^/Hz in the HC group, and 2653.7 ± 918.4 μV^2^/M^4^/Hz, 1863.0 ± 578.7 μV^2^/M^4^/Hz, 552.9 ± 1083.1 μV^2^/M^4^/Hz, 384.4 ± 1109.2 μV^2^/M^4^/Hz, and 2638.6 ± 669.1 μV^2^/M^4^/Hz in the MCI group. The other results are shown in Additional file 1. The SMN consists of the bilateral superior parietal lobes, frontal lobe δ activity, and bilateral superior parietal lobe α activity in an anticorrelated state. The use of this network was lower in the MCI group (*t* =  − 0.845, *p* < 0.05, one-tailed test). The memory perception network consisted of bilateral occipital and right parietal lobe δ activity that was anticorrelated with the frontal lobes, θ activity in the parietal lobes and left temporoparietal junction, and α activity in the right temporal lobe. The use of this network was lower in the MCI group (*t* =  − 0.955, *p* < 0.05, one-tailed test). The posterior DMN consisted of bilateral precuneus and temporal theta activity, and the bilateral temporal θ activity was anticorrelated. The use of this network was lower in the MCI group (*t* =  − 0.996, *p* < 0.05, one-tailed test). The VAN consisted of γ activity in the right occipital to inferior parietal and frontal lobes and γ-anticorrelated activity in the right superior parietal and left frontal lobes. The use of this network was lower in the MCI group (*t* =  − 1.712, *p* < 0.05, one-tailed test). The DAN consisted of bilateral β and γ activity in the bilateral superior parietal lobes and anticorrelated β activity in the right temporoparietal junction. The use of this network was lower in the MCI group (*T* =  − 0.868, *p* < 0.05, one-tailed test) (Table 2).

**Table 2:** Mean rs-EEG network activities in HC and MCI group

Variable	Total	HC	MCI	*t* value
	*n* = 449	*n* = 402	*n* = 47	
SMN, μV^2^/M^4^/Hz (mean ± SD)	2749.2 ± 820.1	2760.3 ± 808.3	2653.7 ± 918.4	− 0.845^‡^
Memory perception network, μV^2^/M^4^/Hz (mean ± SD)	1948.0 ± 646.8	1958.0 ± 654.2	1863.0 ± 578.7	− 0.955^‡^
Posterior DMN, μV^2^/M^4^/Hz (mean ± SD)	690.6 ± 1004.1	706.8 ± 994.7	552.9 ± 1083.1	− 0.996^‡^
VAN, μV^2^/M^4^/Hz (mean ± SD)	663.5 ± 1186.1	696.1 ± 1191.8	384.4 ± 1109.2	− 1.712^‡^
DAN, μV^2^/M^4^/Hz (mean ± SD)	2727.5 ± 743.9	2737.9 ± 752.2	2638.6 ± 669.1	− 0.868^‡^

*HC* healthy condition, *MCI* mild cognitive impairment, *SMN* sensorimotor network, *DMN* default mode network, *VAN* ventral attention network, *DAN* dorsal attention network, *SD* standard deviation

^‡^*t* =  − 0.685, for *p* = 0.039

### ERP results

Tables 3 and 4 show the grand average of the latency and amplitude of the ERP waveforms at N2 and P3 for the HC and MCI groups at the Fz, Cz, and Pz electrodes in the congruent, incongruent, and no response conditions, respectively. The median (IQR) of N2 amplitude at the Cz electrode in the no response condition was − 10.3 μV (− 15.9 to − 6.72) in the HC group and − 6.67 μV (− 11.4 to − 4.1) in the MCI group. Wilcoxon tests showed that the N2 amplitude was significantly smaller in the MCI group than in the HC group for the no response condition (*p* < 0.001).

**Table 3:** The latency of the ERP waveforms at N2 and P3

Variable	Total	HC	MCI	*p* value
	*n* = 449	*n* = 402	*n* = 47	
**Congruent condition**				
N2, ch. Fz—latency, ms (IQR)	188 (168–214)	188 (168–214)	188 (171–216)	0.927
N2, ch. Cz—latency, ms (IQR)	200 (178–218)	200 (178–218)	200 (172–218)	0.514
N2, ch. Pz—latency, ms (IQR)	200 (180–216)	200 (180–216)	200 (179–214)	0.745
P3, ch. Fz—latency, ms (IQR)	424 (385–490)	424 (386–490)	430 (370–484)	0.707
P3, ch. Cz—latency, ms (IQR)	428 (388–482)	428 (388–480)	450 (366–502)	0.531
P3, ch. Pz—latency, ms (IQR)	420 (374–471)	420 (377–470)	438 (364–502)	0.497
**Incongruent condition**				
N2, ch. Fz—latency, ms (IQR)	186 (164–210)	184 (164–208)	200 (177–217)	0.011
N2, ch. Cz—latency, ms (IQR)	198 (176–216)	198 (176–216)	196 (175–216)	0.838
N2, ch. Pz—latency, ms (IQR)	198 (178–214)	198 (178–214)	202 (181–215)	0.501
P3, ch. Fz—latency, ms (IQR)	442 (394–496)	440 (392–496)	462 (406–500)	0.389
P3, ch. Cz—latency, ms (IQR)	454 (394–512)	454 (396–514)	450 (380–509)	0.467
P3, ch. Pz—latency, ms (IQR)	436 (368–512)	438 (369–516)	422 (360–481)	0.182
**No response condition**				
N2, ch. Fz—latency, ms (IQR)	192 (170–214)	192 (168–214)	196 (183–220)	0.047
N2, ch. Cz—latency, ms (IQR)	196 (176–216)	198 (176–214)	190 (174–220)	0.963
N2, ch. Pz—latency, ms (IQR)	194 (176–210)	194 (176–210)	194 (174–212)	0.938
P3, ch. Fz—latency, ms (IQR)	506 (430–568)	506 (429–566)	524 (466–577)	0.112
P3, ch. Cz—latency, ms (IQR)	506 (440–564)	510 (440–564)	496 (423–557)	0.231
P3, ch. Pz—latency, ms (IQR)	466 (384–540)	464 (387–536)	476 (351–555)	0.912

*HC* healthy condition, *MCI* mild cognitive impairment, *IQR* interquartile range, *ch.* channel, *ms* millisecond

**Table 4:** The amplitude of the ERP waveforms at N2 and P3

Variable	Total	HC	MCI	*p* value
	*n* = 449	*n* = 402	*n* = 47	
**Congruent condition**				
N2, ch. Fz—amplitude, μV (IQR)	− 5.12 (− 9.44 to − 2.17)	− 5.12 (− 9.43 to − 2.30)	− 5.04 (− 11.76 to − 1.41)	0.900
N2, ch. Cz—amplitude, μV (IQR)	− 6.10 (− 10.39 to − 2.67)	− 6.30 (− 10.63 to − 2.82)	− 4.47 (− 8.35 to − 1.20)	0.068
N2, ch. Pz—amplitude, μV (IQR)	− 8.64 (− 13.54 to − 4.87)	− 8.72 (− 13.61 to − 4.95)	− 7.32 (− 11.44 to − 4.26)	0.209
P3, ch. Fz—amplitude, μV (IQR)	10.69 (7.13–16.92)	10.74 (7.09–16.92)	9.98 (7.18–16.09)	0.573
P3, ch. Cz—amplitude, μV (IQR)	9.88 (6.31–14.04)	9.88 (6.35–14.12)	9.67 (6.12–13.44)	0.593
P3, ch. Pz—amplitude, μV (IQR)	13.86 (9.81–19.92)	14.33 (10.04–20.23)	12.01 (8.36–16.92)	0.030
**Incongruent condition**				
N2, ch. Fz—amplitude, μV (IQR)	− 5.43 (− 9.40 to − 2.58)	− 5.32 (− 9.18 to − 2.44)	− 6.55 (− 10.25 to − 3.78)	0.152
N2, ch. Cz—amplitude, μV (IQR)	− 7.10 (− 10.49 to − 3.47)	− 7.24 (− 10.46 to − 3.49)	− 5.61 (− 11.36 to − 3.09)	0.570
N2, ch. Pz—amplitude, μV (IQR)	− 9.38 (− 14.23 to − 5.63)	− 9.39 (− 14.08 to − 5.68)	− 9.08 (− 15.56 to − 4.82)	0.982
P3, ch. Fz—amplitude, μV (IQR)	10.88 (7.17–15.80)	10.96 (7.26–15.80)	10.62 (5.80–13.96)	0.269
P3, ch. Cz—amplitude, μV (IQR)	9.59 (6.20–13.90)	9.81 (6.42–13.97)	9.01 (5.28–12.54)	0.299
P3, ch. Pz—amplitude, μV (IQR)	12.71 (8.43–18.29)	12.87 (8.73–18.09)	10.18 (7.16–20.15)	0.232
**No response condition**				
N2, ch. Fz—amplitude, μV (IQR)	− 10.08 (− 15.36 to − 5.98)	− 10.13 (− 15.55 to − 5.99)	− 9.75 (− 14.40 to − 5.53)	0.367
N2, ch. Cz—amplitude, μV (IQR)	− 9.95 (− 15.30 to − 6.23)	− 10.29 (− 15.89 to − 6.72)	− 6.67 (− 11.40 to − 4.09)	< 0.001^*^
N2, ch. Pz—amplitude, μV (IQR)	− 10.82 (− 16.18 to − 6.57)	− 11.15 (− 16.73 to − 6.76)	− 8.82 (− 13.88 to − 4.42)	0.010
P3, ch. Fz—amplitude, μV (IQR)	10.79 (5.24–17.75)	11.06 (5.34–18.35)	7.41 (5.02–13.61)	0.047
P3, ch. Cz—amplitude, μV (IQR)	10.20 (6.19–17.16)	10.68 (6.20–17.39)	8.25 (6.06–12.10)	0.074
P3, ch. Pz—amplitude, μV (IQR)	13.01 (8.01–20.16)	13.14 (8.03–20.23)	11.63 (8.14–17.90)	0.364

*HC*, healthy condition; *MCI*, mild cognitive impairment; *IQR*, interquartile range; *ch.*, channel

^*^p < 0.008 Wilcoxon rank sum test after Bonferroni correction

### Correlation analyses

**Fig. 4:**
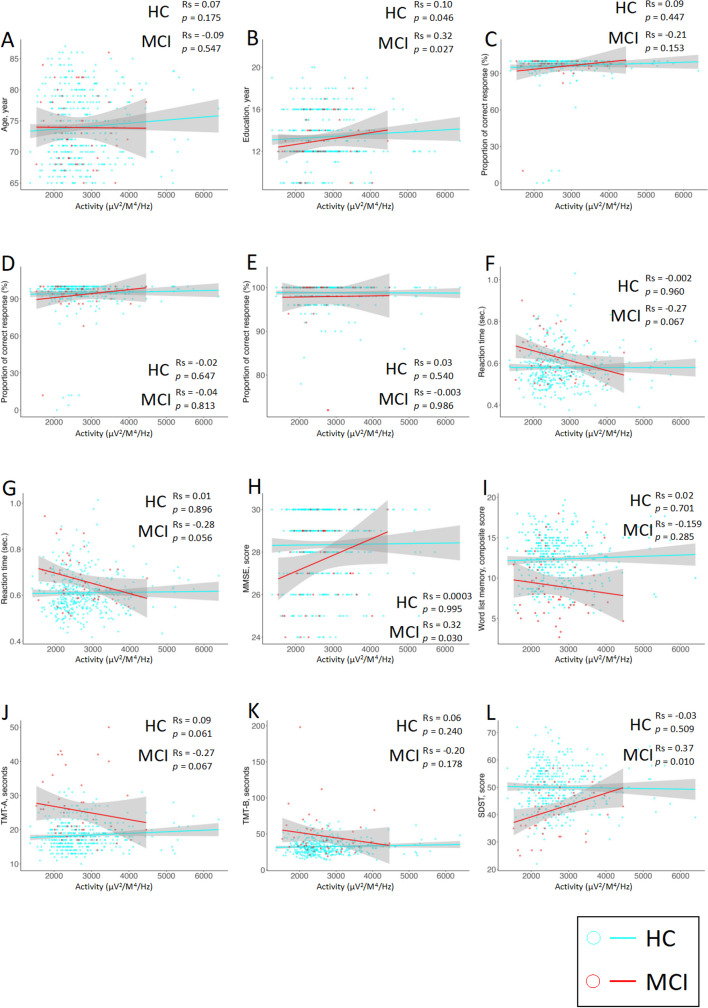
Scatterplot of the DAN (IC-14) activity values with demographic characteristics, cognitive function, and behavioral data. **A** Age. **B** Education. **C** Proportion of correct responses in the congruent condition. **D** Proportion of correct responses in the incongruent condition. **E** Proportion of correct responses in the no response condition. **F** RT in the congruent condition. **G** RT in the incongruent condition. **H** MMSE. **I** Word list memory. **J** TMT-A. **K** TMT-B. **L** SDST

The activities of the DAN (IC-14) which showed correlations with demographic characteristics, cognitive functions, and behavioral data are shown in Fig. 4. Additional results are provided in Additional files 2, 3 and 4. The coefficient (Rs) and *P* value (*p*) of the correlation between DAN and education were Rs = 0.12, *p* = 0.010 in all participants, Rs = 0.10, *p* = 0.046 in the HC group, and Rs = 0.32, *p* = 0.027 in the MCI group. The Rs and *p* of the correlation between DAN, MMSE, and SDST were Rs = 0.04, *p* = 0.441, and Rs = 0.01, *p* = 0.790 in all participants; Rs = 0.0003, *p* = 0.995, and Rs =  − 0.03, *p* = 0.509 in the HC group; and Rs = 0.32, *p* = 0.030 and Rs = 0.37, *p* = 0.010 in the MCI group. The Rs and *p* of the correlation trend between the DAN and the TMT-A and the RTs in the congruent and incongruent conditions, respectively, were Rs = 0.04, *p* = 0.441, Rs =  − 0.04, *p* = 0.457, and Rs =  − 0.03, *p* = 0.594 in all participants; Rs = 0.09, *p* = 0.061, Rs =  − 0.002, *p* = 0.960, and Rs = 0.01, *p* = 0.896 in the HC group; and Rs =  − 0.27, *p* = 0.067, Rs =  − 0.27, *p* = 0.067, and Rs =  − 0.28, *p* = 0.056 in the MCI group.

### Discriminating MCI by SVM and logistic analyses

Random oversampling corrected the imbalance in the numbers of participants in the HC and MCI groups from 402:47 to 234:215. eLORETA-ICA extracted five networks that showed significant differences between the HC and MCI groups: SMN (IC-2), memory perception network (IC-5), backward DMN (IC-7), VAN (IC-11), and DAN (IC-14). A weighted composite score was calculated from the *z*-scores of these. The accuracies in models 1, 2, 3, 4, 5, and 6 were 0.7372, 0.5434, 0.7394, 0.7817, 0.7751, and 0.7929, respectively, in the SVM analysis, and 0.7238, 0.6125, 0.7416, 0.7684, 0.7706, and 0.7795, respectively, in the logistic regression analysis (Table 5). The sensitivities in models 1, 2, 3, 4, 5, and 6 were 0.6605, 0.0465, 0.6465, 0.7209, 0.7070, and 0.7163 in the SVM analysis and 0.6651, 0.3070, 0.6884, 0.7395, 0.7395, and 0.7535 in the logistic regression analysis, respectively (Table 5). The specificities in models 1, 2, 3, 4, 5, and 6 were 0.8077, 1.0000, 0.8248, 0.8376, 0.8376, and 0.8632, respectively, in the SVM analysis, and 0.7778, 0.8932, 0.7906, 0.7949, 0.7991, and 0.8034, respectively, in the logistic regression analysis (Table 5). The precisions of models 1, 2, 3, 4, 5, and 6 were 0.7594, 1.0000, 0.7722, 0.8031, 0.8000, and 0.8280, respectively, in the SVM analysis, and 0.7333, 0.7253, 0.7513, 0.7681, 0.7718, and 0.7788, respectively, in the logistic regression analysis (Table 5). The F1 scores in models 1, 2, 3, 4, 5, and 6 were 0.7065, 0.0889, 0.7038, 0.7598, 0.7506, and 0.7681, respectively, in the SVM analysis, and 0.6976, 0.4314, 0.7184, 0.7536, 0.7553, and 0.7660, respectively, in the logistic regression analysis (Table 5). The AUC in models 1, 2, 3, 4, 5, and 6 were 0.8077, 0.5063, 0.7996, 0.8400, 0.8424, and 0.8495, respectively, in the SVM analysis, and 0.7941, 0.5063, 0.7986, 0.8408, 0.8419, and 0.8492, respectively, in the logistic regression analysis (Table 5).

**Table 5:** Discriminating MCI by SVM and logistic regression analyses

	**Accuracy**	**Sensitivity**	**Specificity**	**Precision**	**F1 Score**	**AUC**
**SVM**						
Model 1	0.7372	0.6605	0.8077	0.7594	0.7065	0.8077
Model 2	0.5434	0.0465	1.0000	1.0000	0.0889	0.5063
Model 3	0.7394	0.6465	0.8248	0.7722	0.7038	0.7996
Model 4	0.7817	0.7209	0.8376	0.8031	0.7598	0.8400
Model 5	0.7751	0.7070	0.8376	0.8000	0.7506	0.8424
Model 6	0.7929	0.7163	0.8632	0.8280	0.7681	0.8495
**Logistic**						
Model 1	0.7238	0.6651	0.7778	0.7333	0.6976	0.7941
Model 2	0.6125	0.3070	0.8932	0.7253	0.4314	0.5063
Model 3	0.7416	0.6884	0.7906	0.7513	0.7184	0.7986
Model 4	0.7684	0.7395	0.7949	0.7681	0.7536	0.8408
Model 5	0.7706	0.7395	0.7991	0.7718	0.7553	0.8419
Model 6	0.7795	0.7535	0.8034	0.7788	0.7660	0.8492

Model 1: Cognitive function tests (Word list memory, TMT-A, TMT-B, and SDST)

Model 2: eLORETA-ICA network activities composite score

Model 3: Combination of Model 1 and Model 2

Model 4: Combination of Model 3 and confounding factors

Model 5: Combination of Model 1, confounding factors, and MRI data

Model 6: Combination of all factors

*SVM* support vector machines, *Logistic* logistic regression, *AUC* area under the curve

## Discussion

Functional abnormalities in the resting brain network have been increasingly reported in patients with MCI [67, 68]. In the present study, MCI was associated with decreased activity of the DAN and decreased amplitude of the N2 component [22, 30, 69, 70]. These findings are consistent with those of previous studies that used rs-MRI, rs-EEG, and ERP. The reduced DAN activity observed in MCI may reflect the neural basis of degenerative top-down attentional deficits [71, 72], as observed in MCI individuals with altered functional anatomy with attenuation of prefrontal cortical activation governing segmental attention [73]. In addition, significantly reduced top-down co-selection has been reported in individuals with MCI, which is further exacerbated in individuals with AD [74]. The decreased amplitude of N2 in MCI individuals is thought to be related to the reduced function of the temporal and parieto-occipital lobes that produce the N2 component, suggesting that the allocation of attentional resources to target stimuli is reduced in MCI individuals [70]. Despite the absence of differences in brain volume between the HC and MCI groups, clear differences in DAN activity and N2 amplitude were observed, suggesting that EEG measurements have potential as neurophysiological markers for detecting preclinical stages of AD in older adults. Moreover, DAN activity correlated with education and cognitive function in patients with MCI. Previous studies indicated that the DAN exhibits an earlier decline in functional connectivity than the VAN [10]. These findings further support the efficacy of EEG for the early detection of MCI and functional assessment in community-dwelling older adults at potential risk for developing AD.

### Simon task and rs-MRI

The present results are consistent with previous studies that reported a reduced proportion of correct responses to Simon tasks and delayed RTs in individuals with MCI [75, 76]. In this study, no significant differences were found in the bilateral hippocampus, CWM, SGM, or TGM between the HC and MCI groups. Given that previous studies have presented the results of systematic reviews of longitudinal studies examining changes in brain structure related to MCI [4], it is possible that the participants in this study were in the early stages of MCI before the brain volume changes began.

### eLORETA-ICA networks

Compared with the HC group, the MCI group showed decreased activity in the SMN, memory perception network, posterior DMN, VAN, and DAN. As a model of graded network degeneration, it is proposed that changes occur in the order of DMN, attention network, and SMN during the transition from preclinical to prodromal AD and AD dementia [77]. In this study, significant differences were found between the HC and MCI groups in the posterior DMN, which is thought to undergo changes earlier than in preclinical AD. The DAN, VAN, and SMN activities, which are also thought to change after preclinical AD, may have been significantly lower in the MCI group. In a study examining the HC and MCI networks using eLORETA-ICA, significant differences were found between the two groups in attention networks [30]. Our study obtained similar results, but the education, MMSE, TMT-A, and TMT-B of the MCI participants in that study were all considerably lower than those in our study. Taken together with the brain volume results, the participants in our study could be considered early MCI participants. Networks similar to ours were suggested to be reduced in MCI in previous fMRI studies [10, 11, 78]. In a previous study comparing the functional connectivity of the DAN and VAN in HC and MCI, the attentional systems in patients with MCI degenerated in a selective manner, specifically with decreased functional connectivity in the DAN but preserved connectivity in the VAN [10, 11]. In this study, the VAN and DAN were significantly decreased in the MCI group compared with the HC group, and the activity of the DAN was significantly correlated with cognitive function. It is possible that changes in the DAN occur at an earlier stage of MCI, suggesting that it may serve as an indicator for early detection of cognitive decline. The results of this study showed that although there were no significant differences in the MRI data between the HC and MCI groups, there were significant differences in several eLORETA-ICA networks. The CR is defined as “a property of the brain that allows for cognitive performance that is better than expected given the degree of life-course related brain changes and brain injury or disease” [79]. We suggest that the eLORETA-ICA network activity revealed in this study is a key factor in maintaining cognitive performance in the face of “life-course related brain changes and brain injury or disease.” Here we suggest that the analysis of rs-EEG data by eLORETA-ICA may be suitable for capturing the neural implementation of CR or the neuroprotective mechanisms of CR. However, this point needs to be investigated in more detail in the future.

### ERP during Simon task

The N2 component is believed to contribute to no-go, conflict, rare target, and stop signals as control-related N2 [16]. In the Simon task used in this study, the participants were taught not to press the button in the no response condition. In the MCI group, the N2 amplitude was lower than in the HC group under the no response condition. This may be the result of capturing control-related N2 components. Previous studies have reported differences in the latency and amplitude of the N2 and P3 components in HC and MCI patients and reported no differences [12]. For example, a series of studies on MCI measured ERP during the Simon task; some reported no difference in the latency of the N2 component in MCI, whereas others reported a difference in the latency and amplitude of the N2 component [21, 70, 80]. The results for the P3 component were similarly mixed, with some studies showing differences depending on the number of cognitive regions in which MCI was impaired [12, 22]. Therefore, further research on ERPs is required after unifying the cognitive tasks used and the types of MCI targeted.

### Correlation between network and demographic characteristics, cognitive function, and behavioral data

Education has been shown to be associated with DAN activity [81], and the results of this study support previous research. Although it has been previously suggested that the DAN is correlated with global cognitive function and processing speed [82, 83], in this study, it was correlated in the MCI group. Several brain networks have been shown to be associated with cognitive function [84], and these networks potentially support each other for complex task demands [85]. In the present study, the MCI group showed reduced activity in the SMN, memory-perception network, VAN, and DAN compared with the HC group. Therefore, the HC group may have been able to use multiple network resources in the cognitive function tests compared with the MCI group. This may explain why the DAN, which was originally shown to be associated with global cognitive function and processing speed, was significantly correlated only in the MCI group.

### Discriminating MCI

The most accurate model for discriminating MCI was based on the following factors: cognitive function tests, composite scores of eLORETA-ICA network activities, MRI data, and confounders. Interestingly, both models, one with eLORETA-ICA network activities added to cognitive tests and confounders and the other with MRI data, showed similarly high accuracy. In a similar study investigating the accuracy of combining rs-EEG and MRI to discriminate HC from MCI, the AUC was moderate, ranging from 0.67 to 0.73 [86]. In the present study, the AUC of the combined EEG and MRI model was 0.8492–0.8495, which is higher than that previously reported. A contributing factor to this difference may be that we used a composite score of eLORETA-ICA network activity, whereas the previous study used a power spectrum analysis of rs-EEG. Our results suggest that the use of rs-EEG instead of rs-MRI may discriminate MCI as well or better. This suggests the efficacy of measuring rs-EEG, which is noninvasive, relatively inexpensive, and less subject to location constraints, and using eLORETA-ICA as an adjunctive neurophysiological marker for discriminating MCI in community-dwelling older adults.

### Limitations

This study had several limitations. First, the sample size of patients with MCI was small compared with that of recent EEG studies (e.g., [30]), and this study did not incorporate long-term follow-up. Thus, whether correlations can be observed longitudinally and cross-sectionally remains to be determined. We also did not assess amyloid and tau levels, which are known to affect neurodegeneration. Several aspects of our method, such as the removal of artifacts and stimulus spacing, were used to optimize the dataset, which likely influenced the results. Therefore, it is important to replicate independent cohorts using pre-specified measures. The Aβ-positivity rate in patients with MCI aged 60–70 years is estimated to be around 50% [87]. This underscores the need to expand the present sample to include a wider range of individuals (e.g., HC, MCI, subjective cognitive decline, and AD) after a complete evaluation of biomarkers. However, the underlying cause of MCI remains unclear. It is known that rs-EEG activity has different topographic features and frequencies depending on whether the MCI is due to neurodegenerative or other diseases [88]. Therefore, the results of this study are not fully understood. In addition, the number of participants with MCI was small and not divided by subtype. In the future, with more participants, we will be able to confirm whether the localization of EEG abnormalities differs when stratified by the MCI subtype. Finally, the correlations among the rs-EEG network, cognitive function tests, and behavioral data must be replicated in an independent data sample with an expanded sample size.

## Conclusions

Although it is known that the clinical symptoms of MCI are a result of neurodegeneration and structural changes in the brain over time due to Aβ and tau deposition, there are currently few studies of how neurophysiological characteristics evaluated using EEG can capture these underlying brain changes and allow early detection of MCI in community-dwelling older adults. Here, we show that the eLORETA-ICA approach to rs-EEG using noninvasive and relatively inexpensive EEG is sensitive to the underlying AD process. It can be used to assess community-dwelling individuals for MCI and may contribute to educational, cognitive function testing, and behavioral data. The rs-EEG measurement is easy to perform and is not subject to the constraints of the measurement environment. It has potential as a neurophysiological marker to detect community-dwelling older adults at risk for the preclinical stages of AD who might otherwise experience delays in seeking medical attention and the detection of cognitive decline and progression.

### Supplementary Information

**Additional file 1.** Mean rs–EEG network activities in HC and MCI group.

**Additional file 2.** Scatterplot of the sensorimotor network (IC–2) activity values with demographic characteristics, cognitive function, and behavioral data. A age; B education; C proportion of correct responses in the Congruent condition; D proportion of correct responses in the Incongruent condition; E proportion of correct responses in the No response condition; F RT in the Congruent condition; G RT in the Incongruent condition; H MMSE; I Word list memory; J TMT–A; K TMT–B; L SDST.

**Additional file 3.** Scatterplot of the memory perception network (IC–5) activity values with demographic characteristics, cognitive function, and behavioral data. A age; B education; C proportion of correct responses in the Congruent condition; D proportion of correct responses in the Incongruent condition; E proportion of correct responses in the No response condition; F RT in the Congruent condition; G RT in the Incongruent condition; H MMSE; I Word list memory; J TMT–A; K TMT–B; L SDST.

**Additional file 4.** Scatterplot of the ventral attention network (IC–11) activity values with demographic characteristics, cognitive function, and behavioral data. A age; B education; C proportion of correct responses in the Congruent condition; D proportion of correct responses in the Incongruent condition; E proportion of correct responses in the No response condition; F RT in the Congruent condition; G RT in the Incongruent condition; H MMSE; I Word list memory; J TMT–A; K TMT–B; L SDST.

## Data Availability

The raw datasets used and/or analyzed during the current study are available from the corresponding author upon reasonable request.

## Abbreviations

Aβ: Amyloid beta
AD: Alzheimer’s disease
ADL: Activities of daily living
AUC: Area under the curve
CR: Cognitive reserve
CWM: Cerebral white matter
DAN: Dorsal attention network
DMN: Default mode network
EEG: Electroencephalography
EHI: Edinburgh Handedness Inventory
eLORETA: Exact low-resolution brain electromagnetic tomography
ERP: Event-related potential
HC: Healthy controls
IC: Independent component
ICA: Independent component analysis
IQR: Interquartile range
MRI: Magnetic resonance imaging
MMSE: Mini-Mental State Examination
NCGG-FAT: National Center for Geriatrics and Gerontology-Functional Assessment Tool
NCGG-SGS: National Center for Geriatrics and Gerontology-Study of Geriatric Syndromes
ROSE: Random Over-Sampling Examples
rs-EEG: Resting-state EEG
rs-fMRI: Resting-state fMRI
RT: Reaction times
SD: Standard deviation
SDST: Symbol digit substitution test
SGM: Subcortical gray matter
SMN: Sensorimotor network
SVM: Support vector machine
TGM: Total gray matter
TMT-A: Trail Making Test Part A
TMT-B: Trail Making Test Part B
VAN: Ventral attention network
Wilcoxon test: Wilcoxon Rank-Sum test
