# Supplementary material for: Neurophysiological markers in community-dwelling older adults with mild cognitive impairment: an EEG study
Source: Alzheimers Res Ther. 2023 Dec 15;15:217. doi: 10.1186/s13195-023-01368-6 (PMC10722716; doi:10.1186/s13195-023-01368-6)
Supplement: Supplementary file 1 — Additional file 1. Mean rs–EEG network activities in HC and MCI group. [file 13195_2023_1368_MOESM1_ESM.docx]

**Additional file 1.** Mean rs–EEG network activities in HC and MCI group

| **Variable** | **Total** | **HC** | **MCI** | ***t* value** |
| --- | --- | --- | --- | --- |
|  | n = 449 | n = 402 | n = 47 |  |
| IC–1, μV^2^/M^4^/Hz (mean±SD) | 2952.7±958.0 | 2959.5±961.4 | 2894.7±936.5 | -0.439 |
| IC–2, μV^2^/M^4^/Hz (mean±SD) | 2749.2±820.1 | 2760.3±808.3 | 2653.7±918.4 | -0.845^‡^ |
| IC–3, μV^2^/M^4^/Hz (mean±SD) | 468.3±1626.1 | 485.7±1621.7 | 319.2±1673.6 | -0.665 |
| IC–4, μV^2^/M^4^/Hz (mean±SD) | 2423.5±664.1 | 2437.2±651.3 | 2306.2±763.3 | -1.284^‡^ |
| IC–5, μV^2^/M^4^/Hz (mean±SD) | 1948.0±646.8 | 1958.0±654.2 | 1863.0±578.7 | -0.955^‡^ |
| IC–6, μV^2^/M^4^/Hz (mean±SD) | -646.0±869.1 | -673.9±820.0 | -408.0±1195.6 | 1.996 |
| IC–7, μV^2^/M^4^/Hz (mean±SD) | 690.6±1004.1 | 706.8±994.7 | 552.9±1083.1 | -0.996^‡^ |
| IC–8, μV^2^/M^4^/Hz (mean±SD) | -2654.9±526.9 | -2654.7±539.1 | -2656.4±412.6 | -0.022 |
| IC–9, μV^2^/M^4^/Hz (mean±SD) | -3133.1±1189.1 | -3135.3±1196.1 | -3113.9±1139.6 | 0.117 |
| IC–10, μV^2^/M^4^/Hz (mean±SD) | -1930.8±939.9 | -1948.0±935.2 | -1783.4±977.1 | 1.139 |
| IC–11, μV^2^/M^4^/Hz (mean±SD) | 663.5±1186.1 | 696.1±1191.8 | 384.4±1109.2 | -1.712^‡^ |
| IC–12, μV^2^/M^4^/Hz (mean±SD) | -470.1±799.0 | -485.6±804.3 | -338.3±746.3 | 1.199 |
| IC–13, μV^2^/M^4^/Hz (mean±SD) | -2735.6±748.6 | -2735.4±758.8 | -2737.1±662.4 | -0.015 |
| IC–14, μV^2^/M^4^/Hz (mean±SD) | 2727.5±743.9 | 2737.9±752.2 | 2638.6±669.1 | -0.868^‡^ |
| IC–15, μV^2^/M^4^/Hz (mean±SD) | 9618.3±734.4 | 9599.0±746.1 | 9783.8±607.6 | 1.639 |

HC: Healthy condition; MCI: Mild Cognitive Impairment; SD: Standard deviation.

^‡^t=-0.685, for p=0.039
