# Supplementary material for: Neurophysiological markers in community-dwelling older adults with mild cognitive impairment: an EEG study
Source: Alzheimers Res Ther. 2023 Dec 15;15:217. doi: 10.1186/s13195-023-01368-6 (PMC10722716; doi:10.1186/s13195-023-01368-6)
Supplement: Supplementary file 3 — Additional file 3. Scatterplot of the memory perception network (IC–5) activity values with demographic characteristics, cognitive function, and behavioral data. A age; B education; C proportion of correct responses in the Congruent condition; D proportion of correct responses in the Incongruent condition; E proportion of correct responses in the No response condition; F RT in the Congruent condition; G RT in the Incongruent condition; H MMSE; I Word list memory; J TMT–A; K TMT–B; L SDST. [file 13195_2023_1368_MOESM3_ESM.docx]

**Additional file 3** Scatterplot of the memory perception network (IC–5) activity values with demographic characteristics, cognitive function, and behavioral data. **A** age; **B** education; **C** proportion of correct responses in the Congruent condition; **D** proportion of correct responses in the Incongruent condition; **E** proportion of correct responses in the No response condition; **F** RT in the Congruent condition; **G** RT in the Incongruent condition; **H** MMSE; **I** Word list memory; **J** TMT–A; **K** TMT–B; **L** SDST.
